# Predicting Institutional Violence: Utility of the Psychopathy Checklist-Revised in a Mexican Prison Context

**DOI:** 10.1177/10731911251368062

**Published:** 2025-10-02

**Authors:** Annalena Schmid, Eric García-López, Barry Rosenfeld, Alicia Nijdam-Jones

**Affiliations:** 1 University of Manitoba, Winnipeg, Canada; 2 Maastricht University, The Netherlands; 3 Instituto Nacional de Ciencias Penales, Mexico; 4 Fordham University, Bronx, NY, USA

**Keywords:** Mexico, psychopathy checklist-revised, predictive utility, cross-cultural validity, institutional violence

## Abstract

Given the high prevalence of institutional violence within the Mexican prison system, the need for validated risk assessment measures is urgent. However, research on the predictive validity of such tools has been limited mainly to White, Educated, Industrialized, Rich, and Democratic samples. This prospective study used quantitative methods to examine the effectiveness of the Psychopathy Checklist-Revised (PCL-R) in predicting institutional violence in a sample of incarcerated individuals in Mexico over 3 months. Data were collected through semi-structured interviews and prison record reviews from 114 adult males in a medium-security prison in Mexico City. Results showed that the PCL-R total score, Factor 2, and Facets 1, 3 and 4 were significant predictors of institutional violence. These findings have practical implications for risk assessment and management within Mexican correctional populations. Recommendations are offered to enhance the methodological rigor of future research endeavors in this area.

## Introduction

The National Prison Administration in Mexico documented 230,730 incarcerated individuals at the end of February 2023, making it the country with the largest prison population in Central America (Comisión Nacional de Seguridad, 2023). In 2021, there were a total of 1,477 violent incidents across 270 penitentiary institutions recorded (Comisión Nacional de los Derechos Humanos, 2021), highlighting the seriousness of institutional violence in Mexican prisons. The counted incidents included homicide (39), suicide (107), assault (1,253), riots (7), escapes (11), hunger strikes (17), torture and/or mistreatment (1), and abuse (42). Moreover, the Mexican National Survey of Imprisoned Population (Instituto Nacional de Estadística y Geografía, 2021) revealed that of 67,584 adult individuals incarcerated in federal and state prisons, 14.4% felt unsafe in their cells, and 25.9% felt unsafe in the prison environment. Nearly 35% reported being victims of institutional misconduct, which included theft of personal belongings (87.7%), physical assault (25.7%), extortion (18.4%), threats (17.4%), sexual harassment (4.6%), and sexual assault and/or rape (1.2%). These high rates of victimization suggest that there is an urgent need to identify risk assessment approaches to inform strategies to reduce, mitigate, and manage institutional violence within the Mexican criminal legal system.

Within any correctional setting, institutional violence pertains to actual, attempted, or threatened harm toward another person, including physical, verbal, and sexual aggression (Gadon et al., 2006). In addition to an increased risk of extended incarceration for individuals in prison, violent behavior produces proximal economic costs for prison employees, including disability and loss of experienced staff, as well as other more distal consequences, such as a reduction in morale and motivation of key staff (Gadon et al., 2006). Most importantly, institutional violence can seriously injure both individuals in custody and staff. Therefore, assessing an individual’s risk of future institutional violence is crucial to preventing violent incidents and can be used to inform risk management decisions regarding security levels, living conditions, and treatment recommendations.

### Predictive Utility of the PCL-R for Institutional Violence

The association between psychopathy and criminality has been a long-debated issue. A recent umbrella review suggests that psychopathy is robustly linked with dangerousness and is highly relevant for understanding both institutional violence and general violence (Gillespie et al., 2023). This suggests that psychopathy is important not only for predicting violence but also for shaping risk management strategies and improving institutional safety. Psychopathy is most commonly assessed with the Psychopathy-Checklist Revised [PCL-R] (Hare, 1980, 1991, 2003), a clinical tool developed to operationalize the construct of psychopathy. Over time, the PCL-R has evolved into the most widely used and thoroughly validated assessment of psychopathy worldwide (Hare et al., 2018). While research consistently shows strong associations between PCL-R scores and various violence outcomes, the tool has also become increasingly relevant for violence risk assessment and management in forensic and correctional settings (DeMatteo & Olver, 2022; Patrick, 2018). A review of nine surveys on the use of violence risk assessment tools in the United Kingdom and the United States found that the PCL-R, alongside the Historical-Clinical-Risk Management-20 [HCR-20] (Douglas et al., 2014), is the most commonly used instrument for violence risk assessment among forensic practitioners (Hurducas et al., 2014). However, the relationship between psychopathy and institutional violence has yielded mixed empirical findings (Guy et al., 2005; Edens et al., 2001), suggesting that more research is needed to clarify this connection.

The debate regarding the PCL-R’s ability to predict institutional violence has been shaped by contrasting views from DeMatteo et al. (2020a, 2020b), Olver et al. (2020), and Hare et al. (2020). DeMatteo et al. (2020a) expressed concerns about an over-reliance on the PCL-R in assessing institutional violence risk, particularly across different populations. In response, Olver et al. (2020) cited meta-analytic evidence showing the consistent effectiveness of the PCL-R in predicting institutional violence across various settings, a position supported by Hare et al. (2020), who highlighted the tool’s strong empirical validation. While additional factors should be considered, the authors emphasized that the PCL-R remains a valuable tool in forensic assessments. Furthermore, a recent review by DeMatteo and Olver (2022) confirmed small to medium effect sizes for predicting institutional violence (*d* = 0.28–0.54).

To better understand the links between the PCL-R and institutional violence, it is essential to explore the distinct associations of the PCL-R’s factors and facets with future violence to better inform risk assessment and management. Hare (2003) proposed a two-factor model for psychopathy, wherein Factor 1 encompasses the interpersonal and affective aspects with the two corresponding facets, and Factor 2 encompasses social deviance with two additional facets related to lifestyle and antisocial behavior. By understanding which factors of psychopathy are more strongly associated with violent behavior, authorities can enhance their risk assessment and management strategies. This knowledge allows for a more targeted approach to addressing individuals’ specific needs and risks within correctional facilities with the help of the PCL-R.

Current research generally indicates that Factor 2 of the PCL-R is a stronger predictor of institutional violence compared to Factor 1 (Abbiati et al., 2019; Campbell et al., 2009; Folino, 2015; Kennealy et al., 2010; Olver & Wong, 2015; Walters, 2003, 2012). Several meta-analyses support this finding, demonstrating that Factor 2 consistently predicts institutional aggression more effectively than Factor 1 (Kennealy et al., 2010; Leistico et al., 2008; Walters, 2003, 2012; Walters et al., 2008; Walters & Heilbrun, 2010). Moreover, a study investigating the differential associations of the single facets of the PCL-R with institutional violence (Walters et al., 2010) identified the antisocial facet (Facet 4) of the PCL-R as the most influential predictor of institutional aggression. In fact, the inclusion of Facet 4 in the prediction model diminished the predictive utility of the other three facets. This finding underscores the significant role of the antisocial behaviors captured by Factor 2 in understanding institutional violence.

While the predictive ability of Factor 2 for institutional violence is strongly supported, the relevance of Factor 1 traits in assessing violence risk should not be dismissed. Some literature suggests that the interpersonal and affective traits captured by Factor 1 may be more strongly associated with certain forms of violence, particularly instrumental violence. For example, Blais et al. (2014) found that the interpersonal facet (Facet 1) of Factor 1 had stronger links to instrumental violence, while the lifestyle facet (Facet 3) of Factor 2 was more closely associated with reactive violence. Since institutional violence can involve both instrumental and reactive aggression, neither dimension of the PCL-R can be considered categorically more relevant for risk assessment.

There is little to suggest that PCL-R total scores become more or less predictive based on how Factor 1 and Factor 2 items are weighted. Instead, DeMatteo and Olver (2022) recommend using the PCL-R alongside additional risk assessment tools to develop comprehensive risk formulations, while also reporting factors and facet scores. This approach highlights that while Factor 2’s association with antisocial behavior and impulsivity is crucial for understanding certain types of institutional violence, Factor 1 traits remain important. Supporting this, Olver et al. (2020) found that Factor 1 was predictive of a latent risk variable that included violence among other behaviors. These findings emphasize the need for a nuanced risk assessment strategy that integrates both Factor 1 and Factor 2, recognizing that individuals high in Factor 1 traits may require different management strategies compared to those whose violence is more impulsive and antisocial.

### Cross-Cultural Validity of the PCL-R

While scholars continue debating the usefulness of the single PCL-R factors and facets in predicting institutional violence, research overlooks one common component: cross-cultural validity. Most research utilizes White, Educated, Industrialized, Rich, and Democratic samples (Henrich et al., 2010), and tools used to predict institutional infractions have been normed almost exclusively on White Americans of European heritage in Canada and the United States (Sullivan & Kosson, 2006). Thus, the scientific evidence of cross-cultural predictive validity of risk assessment measures remains largely unclear (Shepherd & Lewis-Fernandez, 2016). As a result, if these tools are less accurate for certain cultural groups, individuals within those groups might face disadvantages. This is because information from risk assessments greatly influences individuals’ freedoms, given that treatment and risk management decisions often rely on these instruments (Shepherd & Lewis-Fernandez, 2016).

Importantly, limited cross-cultural validity stands in contrast to ethical guidelines and principles (American Psychological Association [APA], 2017a, 2020), and forensic assessments are required to be informed by general guidelines for working in culturally informed ways (APA, 2017b). It seems notable within the scope of this article to specifically highlight that psychologists who conduct psychological testing, assessment, and evaluation are required to consider the unique issues that may arise when test instruments and assessment approaches designed for specific populations are used with diverse populations (APA, 2020, p. 20). This is important due to multiple potential types of biases that occur in the evaluation of risk across diverse populations. Cooke and Hart (2017) outline four types of common biases that may be introduced into the risk assessment process, including conceptual invariance (differential relevance of a concept for risk across cultures), structural invariance (differential makeup of risk factors across cultures), metric invariance (differential meanings of scores on risk assessment tools across cultures), and predictive invariance (equivalence or difference in the predictive ability of risk assessment tools across cultures). To account for those biases, models such as the Race-Informed Forensic Mental Health Assessment (Ratkalkar et al., 2023) have been suggested to enhance cultural competence among practitioners. Yet, despite the theoretical considerations and practical guidelines that have been published over the past decade, no clear consensus exists on best practices. This leads to challenges in conducting culturally informed evaluations, including a lack of appropriate assessment tools, a lack of guidelines, a lack of colleagues from diverse backgrounds, and a lack of relevant research (Fanniff et al., 2022).

Existing research on the cross-cultural validity of the PCL-R primarily focuses on diverse samples within North American correctional and forensic settings. For example, Walsh (2013) compared the predictive validity of the PCL-R for violence among a total of 424 European Americans, African Americans, and Latin Americans incarcerated in a jail in the United States. Notably, the group of Latin Americans was the only group where the PCL-R total score was not predictive of violence. The only facet predictive of violence in this group was the antisocial facet. The author concluded that the predictive utility of the PCL-R did not show equivalent empirical support for different cultural groups, and specifically not for the sample of Latin American individuals incarcerated in the United States. While studies like Walsh’s are valuable, it is crucial to stress the importance of directing studies on the cross-cultural predictive accuracy of risk assessment tools toward local populations. The need for culturally appropriate assessment tools that account for demographic factors relevant to test performance is emphasized in standard guidelines (American Educational Research Association et al., 2014; APA, 2010). This will inform local practitioners on the reliability and validity of risk assessment tools they would like to use with their clients and serve as a reference to adapting existing tools to integrate cultural factors (Bergkamp et al., 2023). Consequently, the risks and needs of individuals from a particular cultural group can be safely managed without disregarding their cultural identity.

Only a few studies have addressed the applicability of the PCL-R in predicting institutional violence in Latin American correctional and forensic populations. The tool has been translated and adapted to a Spanish version (Torrubia et al., 2010) and received support for a similar factor structure to the original PCL-R in samples from Mexico, Colombia, Brazil, and Chile (Acosta et al., 2008; Flores-Mendoza et al., 2008; León-Mayer et al., 2015; Ostrosky-Solís & Ortega, 2008). Nevertheless, research on its predictive validity for future violence in institutional settings is scarce. One study by Folino (2015) assessed the predictive utility of the PCL-R in an Argentinian sample of 153 males pre-released following criminal convictions. Consistent with studies utilizing White, Educated, Industrialized, Rich, and Democratic samples, the author observed slightly improved outcomes of Facets 3 and 4 (AUC = .60, AUC = .62 for general recidivism, and AUC = .61, AUC = .64 for violent recidivism, respectively) when compared to Facets 1 and 2 (AUC = .53, AUC = .53 for general recidivism, and AUC = .51, AUC = .53 for violent recidivism, respectively). This observation was made with an average follow-up time of 1,290 days after release into the community. To our knowledge, no study has been conducted on the predictive validity of the PCL-R for institutional violence in Mexican correctional samples. Hence, there is a need to establish the validity of the PCL-R in institutional populations in Mexico to promote cultural sensitivity when using the tool for the assessment and management of violence risk (Shepherd & Lewis-Fernandez, 2016). This is important not only for applying the tool in local populations but also with recent Mexican immigrant populations in other countries.

### Hypotheses, Aims, and Objectives of the Current Study

The lack of studies on the PCL-R’s predictive validity for institutional violence with Spanish-speaking Latin American samples raises concerns about the cross-cultural utility of the tool. To fill in this research gap, the purpose of our study was to examine the ability of the Spanish version of the PCL-R to predict institutional violence utilizing a sample of adult males incarcerated in a Mexican medium-security prison. Specifically, our study aimed to investigate the predictive validity of the PCL-R total score and its factors and facets for institutional violence. Based on findings in previous studies, it was hypothesized that Factor 2 and its facets would provide a better prediction of institutional violence than Factor 1 and its facets and the PCL-R total score. Furthermore, we aimed to explore the incremental validity effects of the PCL-R total score, its factors, and facets when controlled for age, which has proven to be a robust predictor of future violence.

## Method

### Transparency and Openness

Data for this study were collected in 2018 and 2019, and it was not preregistered. Our internationally diverse team, comprising members from Mexico, Germany, Canada, and the United States, facilitated broad perspectives, cultural sensitivity and considerations in the research process, access to diverse resources, and cohesive research output. This study is part of a larger project on the cross-cultural use of forensic assessment measures by Nijdam-Jones et al. (2021, 2022; Cortvriendt et al., 2024). A de-identified copy of the dataset used and analyzed for the current study is available from the senior author on reasonable request. We report how we determined our sample size, all data exclusions, all manipulations, and all measures in the study.

### Participants

Data were collected from 114 individuals incarcerated in three different prison units in a medium-security prison (El Reclusorio Varonil Preventivo Oriente) in Mexico City between November 2018 and July 2019. One unit housed men who had an increased privilege level and worked within the prison, another unit housed individuals who had more extensive juvenile records, and the third unit housed individuals who were reported to have no previous behavioral rule infractions in prison and higher levels of education, as well as the LGBTQIA+^1^ population. Individuals were included in the study if they spoke Spanish fluently, were over 18 years of age, and had been incarcerated for at least one month before participating. Individuals were excluded from the study if their release was scheduled within four months of the research interview, as this would impact follow-up data collection.

A total of 194 individuals attended one of four information sessions, of which 144 consented to participate in the study. The final research interviews were completed by 128 participants. In total, 14 participants were excluded from the study due to transfer to other facilities, early release, or unavailability for the follow-up date. All remaining 114 participants were male and Mexican-born. Their age ranged from 20 to 66 years (*M* = 36.86 years, *SD* = 9.93 years), and their education level ranged from zero to 17 years (*M* = 8.21 years, *SD* = 2.95 years). Most participants identified as heterosexual (*n* = 109, 96.5%), and more than half of the sample reported being married or cohabitating (*n* = 59, 52.2%) at the time of incarceration. Previous mental health treatment was reported by 21 (18.6%) participants, and experience of clinical symptoms in the six months before the research interview was endorsed by 64.9% (*n* = 74) of the sample. Most of the participants reported a history of substance abuse problems (*n* = 69, 62.2%). Participants had been incarcerated for an average of 56.94 months (*SD* = 40.58 months) before the research interview. Most of the participants had been previously incarcerated (*n* = 74, 64.8%) and convicted (*n* = 107, 93.9%). Participant’s current charges included violent offenses (*n* = 100, 87.6%): homicide or attempted homicide (*n* = 15, 13.3%), sexual offenses (*n* = 19, 16.8%), kidnapping or human trafficking (*n* = 20, 17.7%), and other violations, including violence or threat of violence (*n* = 45, 39.8%). A minority of participants were incarcerated for nonviolent offenses (*n* = 14, 12.4%). In 2021, around 60% of sentences for Mexico’s incarcerated population were for violent crimes, including homicide, assault, sexual assault, and kidnapping (Instituto Nacional de Estadística y Geografía, 2021). The reported 60% refers specifically to primary sentencing offenses. However, some non-violent offenses, such as theft or illegal firearm possession, may still involve violent elements. In the present study, the inclusion criterion requiring a minimum of three months remaining on the sentence may have introduced a selection bias, potentially underrepresenting non-violent offenders with shorter sentences.

### Measures

#### Psychopathy Checklist-Revised

The PCL-R (Hare, 1980, 1991, 2003) is a clinician-rated assessment scale used to evaluate the presence of psychopathic traits in individuals. The checklist consists of 20 items scored on a 3-point scale (0 = *the item does not apply to the individual*, 1 = *the item applies to a certain extent*, and 2 = *the item applies to the individual*; Hare, 1991, 2003), capturing an individual’s behavior and functioning over the lifetime. All items are distributed on the two factors (interpersonal and affective, and social deviance) and the four facets (interpersonal, affective, lifestyle, and antisocial), except items 11 (promiscuous sexual behavior) and 17 (many short-term marital relationships). The evaluator assigns a categorical score to each of the 20 items, indicating the extent and severity of psychopathic traits in an individual.

Various studies have examined the PCL-R’s psychometric properties and use in forensic and correctional settings. In research settings, studies have shown good internal consistency (α = .85 to .89; Hare et al., 1990) and interrater reliability (ICC = .87; Hare, 2003). However, independent studies have revealed variance in the reliability of the tool in field settings, with a range in interrater reliability from poor (ICC = .45; Edens et al., 2010) to excellent (e.g., ICC = .90 to .92; Ismail & Looman, 2018; Porter et al., 2003).

For this study, PCL-R coders used the Spanish PCL-R and manual, translated and adapted in Spain (Torrubia et al., 2010). Ostrosky-Solís & Ortega (2008) supported a similar factor structure of the translated tool to the original PCL-R in a sample of 144 incarcerated adult males in Mexico, demonstrating good internal consistency for the tool (α = .87). The authors concluded that the psychometric properties of the Spanish PCL-R are consistent with the original English version and that it holds utility with Mexican forensic populations. The sample utilized in the present study revealed acceptable internal consistency (α = .70). The average-measures ICC (absolute agreement, *N* = 20) in a two-way random effects mixed-model yielded excellent interrater reliability according to interpretation guidelines by Koo and Li (2016; ICC = .94, 95% CI [.84, .98]). Interrater reliability for the single factor and facet scores revealed good to excellent results, without exception. The average inter-item correlation for all PCL-R items was *r* = .11, indicating relatively weak associations and predominantly unique variance. For each single facet, the mean inter-item correlations reflected a reasonable level of internal consistency (Facet 1: *r* = .20; Facet 2: *r* = .30; Facet 3: *r* = .16; Facet 4: *r* = .21).

#### Institutional Violence

The outcome variable of institutional violence was collected using the Modified Overt Aggression Scale [MOAS] (Kay et al., 1988) Spanish translation (Escala Modificada de Agresión Manifiesta; Arbach, 2007; Arbach & Andres-Pueyo, 2005). The MOAS consists of four subscales: verbal aggression, aggression against property, autoaggression, and physical aggression. The MOAS was completed by correctional officers, a research team review of institutional documents, and follow-up interviews with participants were conducted approximately three months after the initial interview. Institutional violence was coded as a dichotomous variable (0 = not violent, 1 = violent) using the physical aggression subscale, with score sums of two or higher as indicative of institutional violence: 0 = No physical aggression, 1 = Makes menacing gestures, swings at people, grabs at clothing, 2 = Strikes, pushes, scratches, pulls hair of others (without injury), 3 = Attacks others, causing mild injury (bruises, sprain, welts, etc.), 4 = Attacks others, causing serious injury (e.g., fractures, loss of teeth, deep cuts, loss of consciousness).

Due to inconsistencies across the data sources (e.g., vague dates, limited detail, overlapping reports), the research team adjudicated a binary violence outcome variable (0 = no violence, 1 = violence) based on whether at least one verifiable incident of physical aggression (MOAS severity ≥ 2) was documented during the follow-up period. Incidents were included if one source provided sufficient detail to substantiate both the occurrence and severity of the behavior. As verbal, property, and self-aggression were rarely reported (6.1%, 0.9%, and 1.8%, respectively), we did not analyze these forms of aggression, as doing so would have limited statistical power and introduced noise into the analysis. While we originally intended to analyze more detailed, time-stamped incident data for continuous or multi-domain MOAS scoring, our final approach prioritized reliability over granularity given the limitations of the available sources.

### Procedure

Data were collected by research assistants from Mexico City who were enrolled in local university psychology and criminology programs. All assistants underwent comprehensive training in interview procedures and assessment, ensuring their ability to conduct assessments independently. Measures used by the assessors included the PCL-R, the HCR-20, the Violence Risk Appraisal Guide [VRAG] (Harris et al., 1993, Rice et al., 2013), and the Structured Assessment of Protective Factors for violence risk [SAPROF] (De Vogel et al., 2009, 2012). For the present study, only data collected through the PCL-R are utilized.

Participants were recruited through information sessions, where research assistants read a consent form aloud. The consent contained limits of confidentiality and clarified that participation in the study would not affect conditions such as release date, terms of supervision, medical care, security levels, or living conditions. Following recruitment and informed consent, the research assistants reviewed and coded participants’ institutional documents for past aggressive and violent behavior, criminal history, and past mental health treatment. Subsequently, participants completed interviews conducted by the same research assistant, utilizing a semi-structured interview covering family, social, judicial, and cultural variables. Each interview lasted no more than 2 hr, and participants could withdraw at any point during the study.

Whenever incidents of aggressive behavior occurred, correctional officers were instructed to fill out the MOAS form. During the follow-up interview, participants were invited to speak again to a research assistant to discuss any incidents that occurred after the initial interview. The research assistant then completed one (or more) MOAS form(s) based on the participants’ self-reports. The Reclusorio Oriente Technical Committee and Fordham Institutional Review Board approved the original study; the University of Manitoba Research Ethics Board approved secondary data analysis for the current project.

### Statistical Analyses

All data were screened for missing data, outliers, assumptions, and violations of normality. Of the 128 individuals participating in research interviews for this study, 14 were excluded due to a lack of follow-up data on institutional violence. Thus, statistical analyses were conducted with a total of 114 individuals.

Independent samples *t*-tests were used to examine the PCL-R total score and factor differences between participants who engaged in institutional violence versus those who did not. A discriminant analysis was additionally conducted to examine the differential association of the single facets with the outcome variable. *Receiver Operating Characteristic* (ROC) Area under the curve (AUC) statistics were computed to evaluate the predictive validity of the PCL-R total score, factors, and facets on the dichotomous outcome variable of institutional violence with values of .71 and greater indicating excellent predictive validity, .64 to .71 indicating good accuracy, .55 to .63 indicating fair accuracy, and below .55 indicating poor accuracy (Desmarais & Singh, 2013; Rice & Harris, 2005). Furthermore, logistic regression models controlling for age were performed to test whether the PCL-R total scores, its factors, and facets provided predictive value upon age as a widely established predictor of future violence (e.g., Arbach-Lucioni et al., 2012; Cunningham et al., 2005; Schenk & Fremouw, 2012; Steiner et al., 2014). Results of a G*Power analysis (Faul et al., 2009) indicated that 107 participants were needed to generate a power of .80 (α = .05) to detect an effect size of 0.1 or larger for the logistic regression.

## Results

Of the total sample, 24 (21.1%) participants engaged in institutional violence during the three-month follow-up period, while 90 (78.9%) participants did not. Of those engaging in institutional violence, nine (37.5%) received a score of three, reflecting a physical aggression resulting in mild injuries (Table 1). No participants engaged in violence that resulted in serious injuries. The average PCL-R total score for the sample was 14.11 (*SD* = 5.15) and ranged from 5 to 32. According to the PCL-R manual (Hare, 2003), the average score for individuals who criminally offend is approximately 22, which indicates a rather low average score for the present sample. Only one individual had a score higher than the cutoff of 30, indicative of a high expression of psychopathic traits.

**Table 1.** MOAS Aggression Subscales, Scores, and Primary Data Sources.

			MOAS severity score				Primary data source		
MOAS aggression subscale	Participants (*n*)	Total incidents*	1	2	3	4	Self-report	Prison records	Guard report
Physical aggression^a^	24	37	1	24	13	0	21	12	4
Verbal aggression^b^	7	12	38	6	7	5	6	3	3
Property aggression^c^	1	1	1	0	0	0	1	0	0
Self-aggression^d^	2	4	0	0	2	2	2	2	0
Sexual aggression^e^	1	1	1	0	0	0	0	1	0

*Note.* Severity levels follow Modified Over Aggression Scale [MOAS] scoring guidelines (Kay et al., 1988). A single binary outcome variable “institutional aggression” was adjudicated per participant based on corroborated or clearly substantiated reports of physical aggression scores (≥2). This was due to the low base rates of other types of aggression as well as vague, overlapping, or inconsistently dated and reported aggressive incidents across sources that prohibited analysis of incident frequency.

* Only incidents that met the specified criteria for being coded as present were counted in each category.

a Physical aggression coded *present* if MOAS severity score ≥ 2 in this category.

b Verbal aggression coded *present* if MOAS severity score ≥ 3 in this category.

c Property aggression coded *present* if MOAS severity score ≥ 1 in this category.

d Self-aggression coded *present* if MOAS severity score ≥ 1 in this category.

e Sexual aggression coded *present* if MOAS severity score ≥ 1 in this category.

Results of independent samples *t*-tests (Table 2) indicated that the PCL-R total score was significantly higher for individuals who engaged in institutional violence (*M* = 17.21, *SD* = 4.08) compared to those who did not (*M* = 13.29, *SD* = 5.08), *t*(112) = 3.50, *p* = <.001, *d* = 0.80, 95% CI [0.33, 1.26]. Individuals who engaged in institutional violence also scored significantly higher on Factor 2 (*M* = 8.75, *SD* = 1.98) than those who did not (*M* = 6.00, *SD* = 3.47), *t*(112) = 5.04, *p* = <.001, *d* = 0.85, 95% CI [0.39, 1.32]. No significant differences were found for Factor 1.

**Table 2.** Means, Standard Deviations, and Independent Samples *T*-Tests for PCL-R Factors, and Total Score (*N* = 114).

	Sample (*N* = 114)		Nonviolent (*n* = 90)		Violent (*n* = 24)			
Factor scores	*M*	*SD*	*M*	*SD*	*M*	*SD*	*t*	*d*
1: Interpersonal/affective	6.68	2.76	6.49	2.70	7.38	2.93	1.41	0.32
2: Social deviance	6.58	3.40	6.00	3.47	8.75	1.98	5.04**	0.85
Total score								
PCL-R	14.11	5.15	13.29	5.08	17.21	4.08	3.50**	0.80

*Note.* PCL-R = Psychopathy Checklist-Revised.

** p < .01.

The bivariate correlations between the PCL-R facets and the total score are presented in Table 3. Given the weak to moderate strength of these correlations, multicollinearity is unlikely to have impacted the analyses. A discriminant analysis was conducted to determine which PCL-R facets could best differentiate between individuals who engaged in institutional violence and those who did not. The discriminant function was significant (*Λ*, χ2(4) = .853, *p* = .002) and the eigenvalue associated with this function was .172, with a canonical correlation of *r* = .383, indicating a weak to moderate positive association with the outcome variable. The standardized discriminant function coefficients and the structure matrix were examined to interpret the discriminant function. The strongest predictor of the dichotomous outcome was Facet 3 with a coefficient of *r* = .632, followed by Facet 4 with a coefficient of *r* = .563. Facet 1 showed a coefficient of *r* = .534, and Facet 2 showed the weakest association with a coefficient of *r* = -.309. The discriminant function was able to correctly classify 71.9% of cases. Based on the discriminant function, the classification accuracy for the group that engaged in institutional violence was 75.0%, while the group that did not engage in institutional violence had a classification accuracy of 71.1%.

**Table 3.** Correlations for Study Variables.

	PCL-R Total	PCL-R Facet 1	PCL-R Facet 2	PCL-R Facet 3	PCL-R Facet 4	Inst. violence
PCL-R Total	—					
PCL-R Facet 1	.47*	—				
PCL-R Facet 2	.66*	.22*	—			
PCL-R Facet 3	.68*	−.03	.39*	—		
PCL-R Facet 4	.65*	−.05	.22*	.45*	—	
Inst. Violence	.31*	.15	.05	.29*	.28*	—

*Note*. PCL-R = Psychopathy Checklist-Revised.

* *p* < .05.

**Figure 1.**
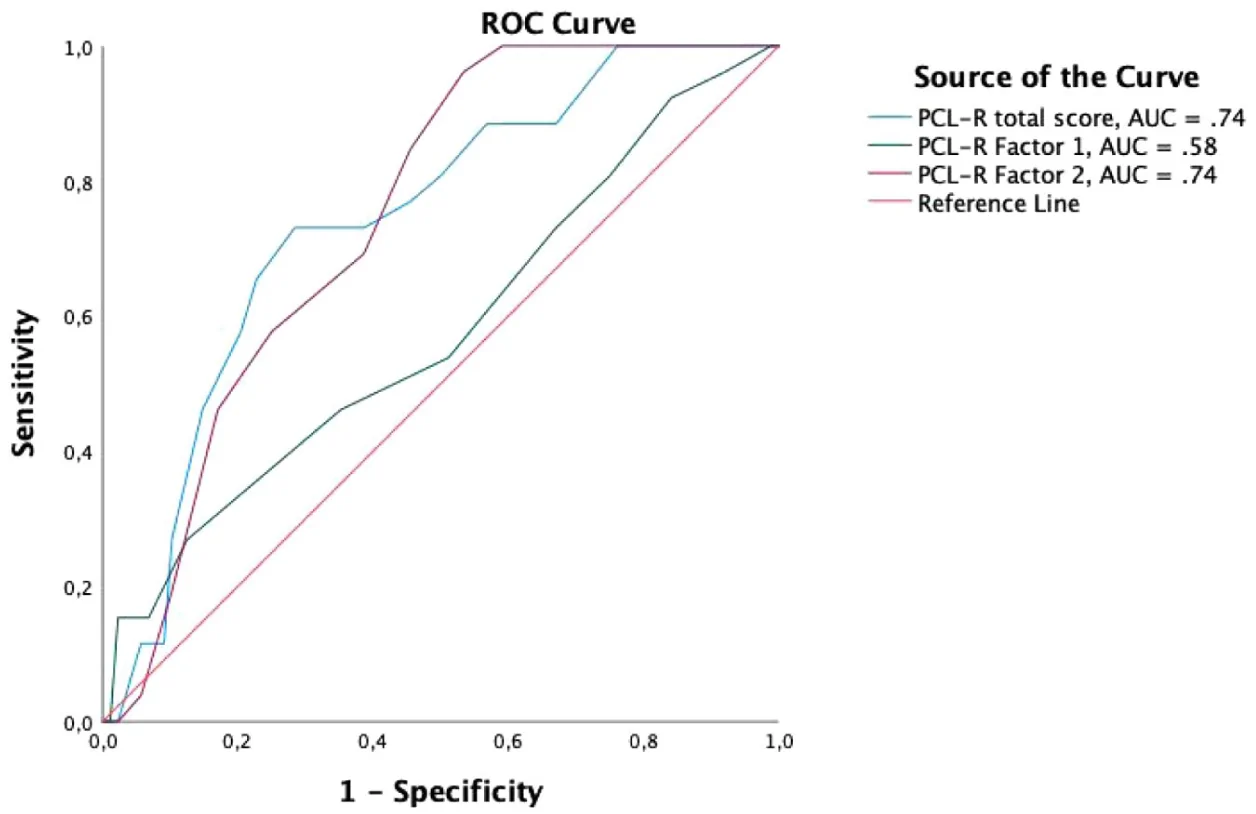
Receiver Operating Characteristic (ROC) curves for PCL-R Total Score, Factor 1, and Factor 2. *Note.* Diagonal segments are produced by ties.

**Figure 2.**
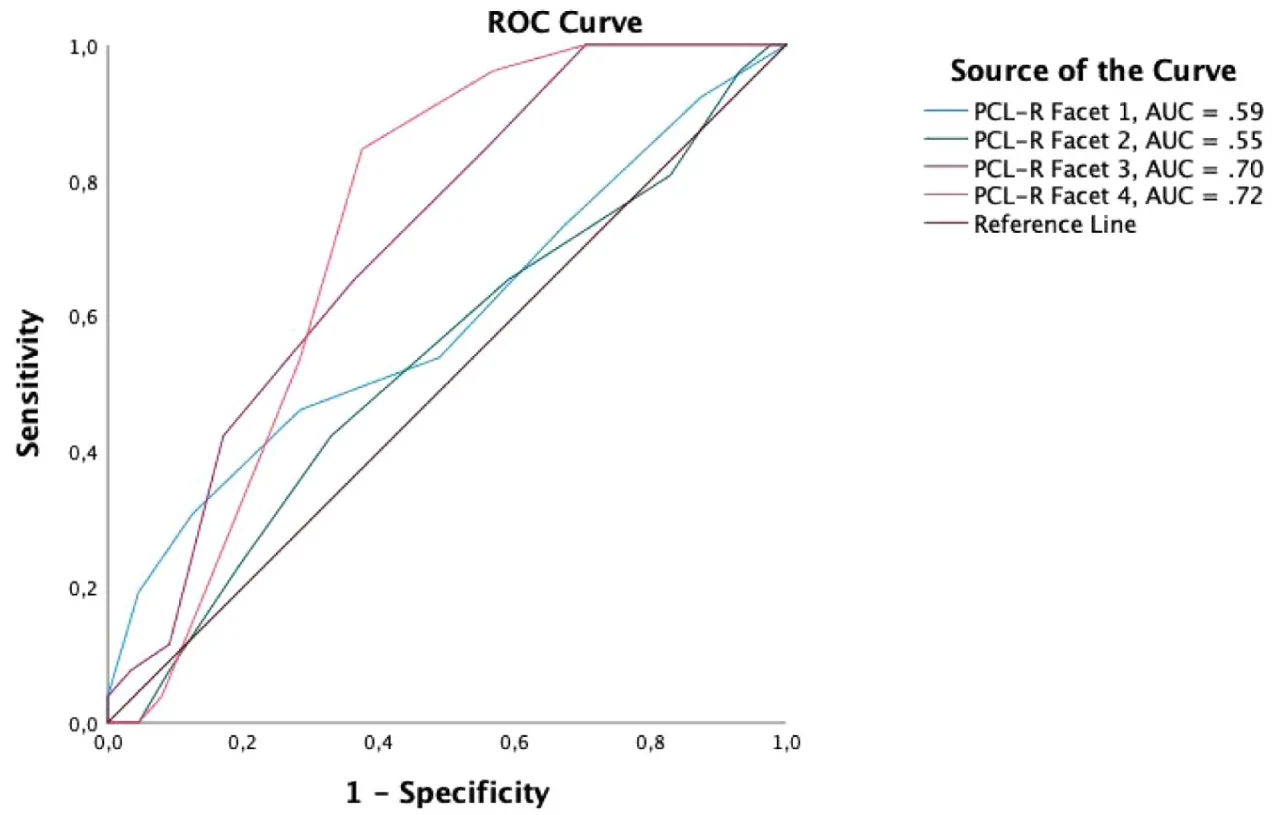
Receiver Operating Characteristic (ROC) curves for PCL-R Total Score and Facets 1–4. *Note.* Diagonal segments are produced by ties.

The PCL-R total score and Factor 2 showed excellent predictive accuracy for institutional violence (AUC = .74, *SE* = .05, 95% CI [.63, .85]; AUC = .74, *SE* = .05, 95% CI [.64, .83], respectively), whereas PCL-R Factor 1 showed fair predictive accuracy (AUC = .58, *SE* = .07, 95% CI [.45, .71]; see Figure 1). Similarly, Facets 3 and 4 showed good and excellent predictive accuracy (AUC = .70, *SE* = .05, 95% CI [.60, .81]; AUC = .72, *SE* = .05, 95% CI [.63, .82], respectively) and Facets 1 and 2 showed fair predictive accuracy (AUC = .59, *SE* = .07, 95% CI [.45, .73]; AUC = .55, *SE* = .07, 95% CI [.41, .68], respectively; see Figure 2), although Facet 2 lies on the cutoff to poor predictive accuracy.

A logistic regression model was conducted to examine the hypothesis that the PCL-R total score would explain a significant amount of the variance in institutional violence in this sample. Results indicated a statistically significant model (see Table 4A), χ^2^(1) = 11.11, *p* < .001, explaining 14.4% of the outcome variance (Nagelkerke *R*^2^) and correctly classifying 77.2% of the cases. When age was included as a predictor (Table 4B), the model was statistically significant, χ^2^(2) = 21.04, *p* < .001, explaining 26.2% of the variance (Nagelkerke *R*^2^) and correctly classifying 79.8% of the cases. Both variables were significant predictors. The likelihood of engaging in institutional violence increased by 0.9 for every one-year older in age (OR = 0.91, 95% CI [0.85, 0.97]) and by 1.17 for every one-unit increase in the PCL-R total score (OR = 1.17, 95% CI = [1.05, 1.30]).

**Table 4.** Logistic Regression Models Predicting Institutional Violence (*N* = 114).

Variables	A. PCL-R Total			B. PCL-R total & Age		
	*B*	OR	95% CI for OR	*B*	OR	95% CI for OR
PCL-R total score	0.16**	1.17	[1.06, 1.29]	0.16**	1.17	[1.06, 1.23]
Age				−0.09**	0.91	[0.85, 0.97]
	C. PCL-R Factors			D. PCL-R Factors & Age		
	*B*	OR	95% CI for OR	*B*	OR	95% CI for OR
Factor 1	0.06	1.07	[0.89, 1.27]	0.10	1.10	[0.91, 1.33]
Factor 2	0.27*	1.31	[1.11, 1.56]	0.24*	1.26	[1.05, 1.52]
Age				−0.09*	0.92	[0.86, 0.98]
	E. PCL-R Facets			F. PCL-R Facets & Age		
	*B*	OR	95% CI for OR	*B*	OR	95% CI for OR
Facet 1	0.30*	1.35	[1.01, 1.81]	0.41*	1.50	[1.09, 2.06]
Facet 2	−0.22	0.80	[0.58, 1.11]	−0.29	0.75	[0.51, 1.10]
Facet 3	0.39*	1.48	[1.07, 2.03]	0.38*	1.47	[1.03, 2.09]
Facet 4	0.31*	1.36	[1.04, 1.78]	0.28	1.32	[0.98, 1.78]
Age				−0.11**	0.90	[0.86, 0.97]

*Note.* Institutional violence coded as 0 = absent and 1 = present.

PCL-R = Psychopathy Checklist-Revised; OR = odds ratio. CI = Confidence Interval. Method: ENTER.

* *p* < .05. ** *p* < .01.

A logistic regression model including Factor 1 and Factor 2 as predictors was statistically significant (Table 4C), χ^2^(2) = 14.13, *p* < .001, explaining 18.1% of the variance (Nagelkerke *R*^2^) and correctly classifying 77.2% of the cases. However, only Factor 2 had a statistically significant effect (*Wald* = 9.74, *p* = .002), while Factor 1 did not add predictive value (*Wald* = 0.49, *p* = .48). The likelihood of engaging in institutional violence increased by 1.31 for every one-unit increase on Factor 2 (OR = 1.31, 95% CI [1.11, 1.56]). When age was included as a predictor, the model was significant (Table 4D), χ^2^(3) = 21.53, *p* < .001, explaining 26.8% of the variance (Nagelkerke *R*^2^) and correctly classifying 78.9% of the cases. Factor 2 remained statistically significant (*Wald* = 6.32, *p* = .01), and Factor 1 remained not statistically significant (*Wald* = 0.1, *p* = .32).

Finally, logistic regression analyses were performed to examine the distinct effects of the four PCL-R facets on institutional violence (Tables 4E & 4F). The variance inflation factor (VIF) for each facet remained within the acceptable range of 0.25 to 4, which indicates that multicollinearity did not likely influence the regression parameters. The model, including all four facets, was statistically significant, χ^2^(4) = 18.71, *p* < .001, explained 23.5% of the variance (Nagelkerke *R*^2^), and correctly classified 80.7% of the cases. All facets added significant value to the prediction, except Facet 2 (*Wald* = 1.73, *p* = .19). The likelihood of engaging in institutional violence, therefore, increased by 1.35 for every one-unit increase on Facet 1 (OR = 1.35, 95% CI [1.01, 1.81]), by 1.48 for every one-unit increase on Facet 3 (OR = 1.48, 95% CI [1.07, 2.03]), and by 1.36 for every one-unit increase on Facet 4 (OR = 1.36, 95% CI [1.04, 1.78]). When age was entered into this model, Facet 4 lost its predictive ability for the outcome variable.

## Discussion

Tools used for violence risk assessment, such as the PCL-R, are largely developed and validated using White, Educated, Industrialized, Rich, and Democratic samples. Thus, the transferability of violence risk assessment research to other global populations is unclear and marks a gap in the literature. We aimed to fill in this gap by examining the predictive validity of the PCL-R for institutional violence in a Mexican prison context. We found that the PCL-R demonstrated excellent predictive validity for institutional violence (AUC = .74), with effect sizes comparable to those of risk assessment tools examined in related studies using the current sample (Cortvriendt et al., 2024; Nijdam-Jones et al., 2021, 2022). Specifically, these studies reported effect sizes of .74 for the VRAG (Cortvriendt et al., 2024) and between .71 and .74 for the HCR-20 (Nijdam-Jones et al., 2021). Therefore, the PCL-R's predictive ability for institutional violence in a Mexican prison sample is on par with, and as reliable as, risk assessment tools specifically designed for this purpose, despite the PCL-R being primarily a clinical measure of psychopathy.

Our results further showed that the PCL-R total score, as well as Factor 2 and its facets were successful at differentiating between individuals who engaged in institutional violence compared to those who did not within a 3-month period. Factor 1 and its facets did not reach statistical significance. This is in line with our hypothesis, which was based on previous findings from various studies and meta-analyses (Abbiati et al., 2019; Campbell et al., 2009; DeMatteo & Olver, 2022; Folino, 2015; Kennealy et al., 2010; Olver & Wong, 2015; Walters, 2003, 2012). Thus, we support the view that Factor 2 has the same magnitude of prediction effect as the total PCL-R score (DeMatteo & Olver, 2022) and propose that this seems to be consistent across culturally and linguistically diverse populations. In the present sample, Factor 2 even showed a slightly larger effect size than the PCL-R total score in differentiating between individuals who engaged in violence versus those who did not. Excellent predictive accuracy was found for the PCL-R total score and Factor 2, as compared to Factor 1, which only showed fair accuracy. Similarly, Facets 3 and 4 showed good and excellent predictive accuracy, respectively, while Facets 1 and 2 both showed fair predictive accuracy. The results suggest that Facet 3 was the strongest predictor of institutional violence, followed by Facet 4. When age was controlled, Facet 1 had the strongest predictive value amongst all facets, and Facet 4 lost its predictive ability. Overall, Facet 2 showed the weakest prediction ability.

An increase in the PCL-R total score by one unit was associated with a 17% rise in the likelihood of engaging in institutional violence. Similarly, a one-unit increase in Factor 2 raised the likelihood of institutional violence by 31%. Again, this suggests a higher predictive value when relying on Factor 2 than the total score for predicting institutional violence, which is in line with previous studies and meta-analyses (Abbiati et al., 2019; Campbell et al., 2009; DeMatteo & Olver, 2022; Folino, 2015; Kennealy et al., 2010; Olver & Wong, 2015; Walters, 2003, 2012). This trend in our sample persisted when age was considered, adding some explanatory power but not significantly enhancing the predictive value of the tool. This stands in contrast to previous research (Olver & Wong, 2015), where the inclusion of age in a prediction model improved its efficacy in predicting recidivism compared to a model with only the PCL-R. As opposed to the longitudinal study conducted by Olver and Wong (2015), the follow-up period in the present study was rather short, which can be part of the reason why age did not provide incremental value upon the prediction of the PCL-R. When individual facets were examined, all except Facet 2 significantly contributed to predictions, with Facets 3 and 4 emerging as the best predictors. Facet 3 stood out as the most valuable predictor of institutional violence, which marks a particularity of the sample being used compared to previous studies using White, Educated, Industrialized, Rich, and Democratic samples, where Facet 4 stands out as the most valuable facet for predicting violence. Interestingly, Facet 4 lost its predictive ability in our sample when age was included in the prediction model. Concurrently, Facet 1 became the most valuable predictor. Implications of this finding will be discussed.

### Clinical Implications and Future Research Directions

Overall, our findings have important implications for the application of the PCL-R as a risk assessment tool and identify the need for more research into its use with culturally and linguistically diverse populations. Previous research on the PCL-R’s predictive validity for institutional violence, such as DeMatteo and Olver (2022), found small to medium effect sizes (*d* = 0.28–0.54). In contrast, our study reports a notably higher predictive power with an AUC of .74 (Rice & Harris, 2005), suggesting that the PCL-R performs more effectively in this specific institutional setting. This finding supports a nuanced view of the PCL-R as both a valuable component of forensic assessments (as argued by Olver et al., 2020) and a tool whose predictive power can vary based on sample and context (as noted by DeMatteo et al., 2020a, 2020b). Overall, while prior research has pointed to moderate effectiveness, our results suggest that the PCL-R’s predictive validity in this sample is comparable to that of specialized violence risk assessment tools.

Our findings also suggest that the four PCL-R facets may vary in their predictive value for institutional violence depending on the cultural context, which could inform culturally responsive risk management and treatment recommendations. While Facet 4 (antisocial) is often identified as the strongest predictor in US and Canadian samples (Walters et al., 2008; Walters & Heilbrun, 2010), we found that Facet 3 (lifestyle) was the most predictive facet in our sample of incarcerated adult males in Mexico when age was not controlled. In the Mexican criminal legal system, violence reduction programs may benefit from targeting lifestyle traits captured by PCL-R Facet 3, such as parasitic lifestyle, lack of realistic goals, and irresponsibility, through job-related training or other skill-building programs. However, while job training may improve responsibility and goal-setting and potentially reduce behaviors linked to institutional violence, it may not fully address the types of violence that are more directly related to power dynamics, such as instrumental violence. Further exploration would be needed to understand how these lifestyle characteristics contribute to specific forms of violence within the prison environment. This understanding would allow for the development of more targeted interventions that not only address the underlying lifestyle issues but also effectively mitigate the specific types of violence most prevalent in this context.

The current study’s results challenge prior assumptions about the importance of PCL-R Factor 1 in predicting institutional violence. Contrary to the belief that Factor 1 might be less relevant, with the tool’s utility being driven mainly by antisocial behaviors captured in Factor 2 (Skeem et al., 2005; Skeem & Cooke, 2010; Skeem & Mulvey, 2001), our findings show that Facet 1 had a significant predictive effect, comparable in magnitude to Facet 4 within our sample. Moreover, when controlling for age, Facet 1 emerged as the strongest predictor of institutional violence across all facets, underscoring its importance in this context. This suggests that Facet 1 may provide valuable predictive information and should not be overlooked. However, it is unclear whether this relationship is driven by our sample’s unique cultural context or if it would be better explained as the result of instrumental aggression. For instance, it is possible that our Facet 1 findings reflect cultural differences in how psychopathic traits are expressed or interpreted in Mexico, potentially suggesting that Facet 1 may hold greater relevance in some Latin American contexts compared to American or Canadian ones. If so, such differences could indicate breaches in structural and predictive invariance of the PCL-R (Cooke & Hart, 2017), underscoring the need for culturally sensitive validation of forensic assessment tools. Alternatively, the predictive value of Facet 1 could also reflect our sample engaging in more instrumental aggression than reactive aggression, as Factor 1 has been more closely related to instrumental aggression in previous investigations (Blais et al., 2014). Unfortunately, our study design did not permit us to distinguish between reactive and instrumental aggression to explore this hypothesis, warranting future research to explore conceptual factors underlying the predictive relationship between Facet 1 and institutional violence.

Our results showed that Facet 4 was no longer significant when age was included in the prediction model, suggesting that age accounted for much of the variance in institutional violence previously explained by antisocial behaviors. This is particularly relevant given the average age in our sample was 36.86 years, which falls beyond the typical peak age range for antisocial conduct described by the age–crime curve (Hirschi & Gottfredson, 1983). Prior research has demonstrated that antisocial behaviors, such as those captured by Facet 4, often decline with age, making age a robust predictor of future violence in some populations (Arbach-Lucioni et al., 2012; Cunningham et al., 2005; Schenk & Fremouw, 2012; Steiner et al., 2014). Thus, the reduced predictive power of Facet 4 in our model may reflect a natural attenuation of antisocial tendencies in our older, Mexican incarcerated sample. These findings highlight the importance of situating PCL-R scores within age-related trends when conducting risk assessments, particularly in populations beyond the peak age of antisociality.

Practitioners should be cautious about relying solely on the PCL-R total score as a predictor for institutional violence without considering the individual factors and facets, particularly in correctional settings like the Mexican criminal legal system. As demonstrated by Blais et al. (2014), the single facets of the PCL-R can have differential explanatory value for different types of institutional aggression. As such, Factor 1 facets seem to be closely related to instrumental aggression as opposed to Factor 2 facets that are more related to reactive aggression. A “high-risk” classification based solely on total PCL-R total scores may misinform clinical, correctional, and legal decision-making, underscoring the need for a nuanced approach. Based on this, we recommend that practitioners adopt a more individualized approach when evaluating the risk of institutional violence, paying close attention to the unique contributions of each PCL-R facet. Given the significant predictive value of Facet 1, alongside Facets 3 and 4, tailored interventions should address the critical factors identified by the PCL-R, enhancing the effectiveness of risk management strategies.

### Strengths and Limitations

Our study employed several robust methodological approaches. First, we ensured that all our research assistants received extensive training before conducting interviews, scoring instruments, and coding variables. To code the PCL-R, we relied on multiple sources of information, such as record reviews and semi-structured interviews. Interrater reliability for the PCL-R total score was excellent, also ranging from good to excellent for the single factor and facet scores. Although the literature suggests that the predictive validity of the PCL-R might be attenuated by the higher scoring subjectivity of Factor 1 and its facets (Miller et al., 2011; Rufino et al., 2011), our results did not indicate significant rater disagreement for any of the factors or facets. Secondly, we used different sources of information to capture incidents of institutional violence during the follow-up period, such as correctional officer reports, official prison records, and participant self-report. This approach helped us limit the possibility of undetected incidents. The findings from our study contribute to the ongoing scholarly discussion about the predictive validity of the PCL-R and its applicability across cultures. The prospective nature of this study design ensured that we collected high-quality data and allowed us to conduct various types of statistical analyses. Still, our results might be skewed due to this sample’s low PCL-R average score. Notably, our study was conducted in an applied setting, enhancing its ecological validity. However, it is important to note that practitioners using these measures in the field would need to undergo extensive training similar to that received by our research assistants. This training is crucial to ensure accurate and reliable application of the measures in practical settings. Due to sampling bias, we acknowledge that our findings cannot be generalized to other institutional settings, different regions in Mexico, other Latin American cultural groups, or Latin Americans living abroad. Furthermore, as participants were self-selected and might differ in their characteristics, attitudes, and behaviors from participants who refused to take part in the study, our sample may not be representative of the broader prison population. To minimize this bias, we suggest that future studies with prison populations employ random sampling methods wherever possible.

It is important to recognize the limitations of our outcome variable. First, we defined institutional violence only in terms of physical aggression. While we recognize the importance of considering different types of aggression, we specifically chose to focus on physical aggression due to its significant impact on victims and its relevance for policymakers. Arguably, physical aggression is often associated with more severe consequences and is more readily measurable compared to other forms of aggression. Furthermore, it is noteworthy that most of the previous studies referenced in our article primarily focused on physical aggression, which, in addition to the low rates of verbal, property and self-aggression reported in our sample, influenced our decision to prioritize this type of aggression in the current study. By acknowledging this limitation, future research can explore a broader range of aggressive behaviors to provide a more comprehensive understanding of institutional violence.

Second, our study assessed institutional violence using a binary measurement approach, which means we did not capture the full range of severity for incidents. Although a strength of the study was the inclusion of multiple sources to document institutional violence, there were still concerns with the quality of the outcome data and whether all instances were captured. In comparison to numerous self-reported incidents of physical or severe verbal aggression, there were only a few documented infractions in participants’ records and even fewer official reports filed by correctional staff. Several factors likely contributed to this discrepancy, including overpopulation, insufficient staffing ratios, and potential corruption within the Mexican prison system (Comisión Nacional de los Derechos Humanos, 2021; Gómez Pérez, 2017; Instituto Nacional de Estadística y Geografía, 2021; Sánchez Galindo, 2011). These contextual limitations may have impacted the reliability and accuracy of violence documentation. In light of these inconsistencies, along with vague dates and limited detail in some reports, violence was coded dichotomously to prevent counting the same incident more than once. Although this approach limited our ability to analyze the frequency or severity of aggression, it provided a conservative and consistent method for capturing clearly substantiated incidents across sources.

Moreover, the proportion of serious aggressive incidents in our sample was notably low. No participant received a score of four for physical aggression, which would indicate physical assault resulting in serious injury, and only nine participants received a score of 3, reflecting physical assault causing mild injuries. This suggests that the majority of participants who engaged in institutional violence in our sample showed less severe forms of aggression (*n* = 15, 62.5%), such as striking or pushing without injury. As a result, our study’s findings may not be generalizable to cases involving serious institutional aggression, which are of greater concern within correctional settings.

Another key limitation of this study is the inability to differentiate between types of institutional aggression, such as instrumental versus reactive violence. Factor 1, particularly Facet 1, is more closely associated with instrumental violence, while Factor 2 and its facets are linked to reactive violence (Blais et al., 2014). This distinction is crucial, as the type of aggression may influence the predictive accuracy of different factors. However, in the present study, we were unable to categorize the type of violence participants engaged in, limiting our ability to fully explore how these factors relate to specific forms of aggression.

Furthermore, the expression of psychopathy and the link between psychopathy and violence may vary across cultural contexts, as behaviors labeled as manipulative or irresponsible in one culture may not carry the same meaning or predictive value in another. It is possible that the unexpected predictive strength of Facet 1 when age is controlled, may reflect such cultural variability. Future research should explicitly test for measurement invariance, including structural, conceptual, and predictive invariance (Cooke & Hart, 2017), to ensure tools like the PCL-R function equivalently across diverse populations.

Finally, due to limited resources and time constraints, our study utilized a short follow-up period; a more extended observation duration would have captured a wider range of violent incidents, enabling a more robust evaluation of the PCL-R’s predictive efficacy for institutional violence. Consequently, there is a need for longitudinal studies to assess long-term predictive validity in this context.

## Conclusions

The results of our study indicate that the PCL-R can be a useful tool in predicting institutional violence in the Mexican criminal legal system with excellent accuracy. Nevertheless, it is important to underscore the need for additional research to validate these findings across diverse cultural and ethnic groups and within varying institutional contexts. Future studies should also incorporate data from risk assessment measures alongside the PCL-R, as this would enable a comparison between clinical and risk assessment tools, enhancing both the conceptual and practical contributions. To strengthen our comprehension of the PCL-R's predictive capabilities, it is crucial to explore its correlations with a broader spectrum of violence-related outcomes, encompassing both violent and non-violent recidivism. While the present study focused on physical aggression in an institutional setting as an indicator of violence, future studies might include other forms of aggression, such as verbal aggression. Overall, if future research allows for more detailed and time-stamped tracking of violent incidents, such data could offer nuanced insights into the temporal dynamics of aggressive behavior and its relationship with psychopathy. Moreover, researchers are encouraged to delve into the distinct effects of the single PCL-R factors and facets in order to identify those with the highest predictive value. This precision is vital for tailoring effective interventions and risk management strategies. Importantly, cross-cultural investigations are essential to unravel variations in the predictive value of different facets, ultimately aiming to establish the PCL-R’s predictive validity for (institutional) violence across diverse cultures.

## Notes

1. Lesbian, gay, bisexual, transgender, queer, intersex, asexual, and others
